# Impact of early in-hospital medication review by clinical pharmacists on health services utilization

**DOI:** 10.1371/journal.pone.0170495

**Published:** 2017-02-13

**Authors:** Corinne M. Hohl, Nilu Partovi, Isabella Ghement, Maeve E. Wickham, Kimberlyn McGrail, Lisa N. Reddekopp, Boris Sobolev

**Affiliations:** 1 Department of Emergency Medicine, University of British Columbia, Vancouver, Canada; 2 Centre for Clinical Epidemiology & Evaluation, Vancouver Coastal Health Research Institute, Vancouver, Canada; 3 Emergency Department, Vancouver General Hospital, Vancouver, Canada; 4 Department of Pharmaceutical Sciences, University of British Columbia, Vancouver, Canada; 5 Coordinator, Clinical Pharmacy Services, Vancouver General Hospital, Vancouver, Canada; 6 Ghement Statistical Consulting Company Ltd, Richmond, Canada; 7 School of Population and Public Health, Vancouver, Canada; 8 Vancouver Coastal Health, Vancouver, Canada; University of Brescia, ITALY

## Abstract

**Background:**

Adverse drug events are a leading cause of emergency department visits and unplanned admissions, and prolong hospital stays. Medication review interventions aim to identify adverse drug events and optimize medication use. Previous evaluations of in-hospital medication reviews have focused on interventions at discharge, with an unclear effect on health outcomes. We assessed the effect of early in-hospital pharmacist-led medication review on the health outcomes of high-risk patients.

**Methods:**

We used a quasi-randomized design to evaluate a quality improvement project in three hospitals in British Columbia, Canada. We incorporated a clinical decision rule into emergency department triage pathways, allowing nurses to identify patients at high-risk for adverse drug events. After randomly selecting the first eligible patient for participation, clinical pharmacists systematically allocated subsequent high-risk patients to medication review or usual care. Medication review included obtaining a best possible medication history and reviewing the patient’s medications for appropriateness and adverse drug events. The primary outcome was the number of days spent in-hospital over 30 days, and was ascertained using administrative data. We used median and inverse propensity score weighted logistic regression modeling to determine the effect of pharmacist-led medication review on downstream health services use.

**Results:**

Of 10,807 high-risk patients, 6,416 received early pharmacist-led medication review and 4,391 usual care. Their baseline characteristics were balanced. The median number of hospital days was reduced by 0.48 days (95% confidence intervals [CI] = 0.00 to 0.96; p = 0.058) in the medication review group compared to usual care, representing an 8% reduction in the median length of stay. Among patients under 80 years of age, the median number of hospital days was reduced by 0.60 days (95% CI = 0.06 to 1.17; p = 0.03), representing 11% reduction in the median length of stay. There was no significant effect on emergency department revisits, admissions, readmissions, or mortality.

**Limitations:**

We were limited by our inability to conduct a randomized controlled trial, but used quasi-random patient allocation methods and propensity score modeling to ensure balance between treatment groups, and administrative data to ensure blinded outcomes ascertainment. We were unable to account for alternate level of care days, and therefore, may have underestimated the treatment effect in frail elderly patients who are likely to remain in hospital while awaiting long-term care.

**Conclusions:**

Early pharmacist-led medication review was associated with reduced hospital-bed utilization compared to usual care among high-risk patients under 80 years of age, but not among those who were older. The results of our evaluation suggest that medication review by pharmacists in the emergency department may impact the length of hospital stay in select patient populations.

## Introduction

Adverse drug events are unintended and harmful events related to medication use, and commonly lead to emergency department visits, unplanned admissions, and prolonged hospital stays.[1, 2] Adverse drug events reduce the treatment benefits of medications, increase drug therapy costs, and are associated with greater inpatient and outpatient health services utilization and costs.[3–7] Reducing preventable adverse drug events and mitigating their impact on health outcomes is a global patient safety priority.[8]

A growing body of literature suggests that physicians commonly do not attribute the signs and symptoms of adverse drug events to medication use both in emergency departments and on hospital wards, leading to missed diagnoses, treatment delays, and re-exposures of patients to culprit medications.[9–13] Medication review is an intervention commonly performed by clinical pharmacists to improve medication safety and health outcomes, and ensure optimal medication use. It involves the critical examination of an individual patient’s medications to identify and resolve medication-related problems, including adverse drug events.[14] To date, only a few randomized trials have evaluated the effect of in-hospital pharmacist-led medication review on mortality, hospital readmission and the number of subsequent emergency department contacts. None have evaluated the effect of medication review performed in the emergency department (*i*.*e*., early medication review) on the subsequent number of days spent in hospital.[15]

In 2011, the British Columbia Ministry of Health and Vancouver Costal Health Authority co-funded a pay-for-performance quality improvement program called the Adverse Drug Event Screening Program. Its aim was to expand access to early in-hospital pharmacist-led medication review for high-risk patients to ensure prompt identification and treatment of adverse drug events, and optimize medications early within the hospital stay. Implementation of the program provided the opportunity to evaluate the effect of early pharmacist-led medication review on downstream health services use.[16] We assessed whether pharmacist-led medication review in the emergency department could reduce the number of days spent in hospital among high-risk patients who were admitted compared to usual care. The evaluation protocol for this study has been published.[16]

## Materials and methods

### Design

This was a pragmatic prospective controlled quality improvement evaluation study that used administrative databases for outcomes ascertainment.

### Setting

The aim of the Adverse Drug Event Screening Program was to expand access to early in-hospital pharmacist-led medication review for high-risk patients to ensure prompt identification and treatment of adverse drug events and to optimize medications early within the hospital stay. The program was implemented between September 2011 and February 2012 in one tertiary care (Vancouver General Hospital) and two urban community hospitals (Lions Gate and Richmond General Hospitals), and was funded to run for 12 months at each site.

### Pilot testing

The Adverse Drug Event Screening Program was implemented in a consecutive manner at participating sites in order for earlier implementation experiences to inform later implementations. The first 8-weeks at each site were a pilot period split into two phases. During the first phase, a nurse educator provided didactic educational sessions and one-on-one feedback to nurses on the application and interpretation of a clinical decision instrument used to identify and flag incoming patients at high-risk for adverse drug events.[17] During the second phase, additional hours of clinical pharmacist, subsequently termed “medication review pharmacist”, coverage were provided to review the medications of high-risk patients. All emergency departments kept their pre-existing emergency department pharmacists, subsequently termed “emergency pharmacists”, who provided one full-time equivalent of pharmacist coverage on weekdays during business hours for *ad hoc* consultations requested by physicians. During the second pilot phase, we determined that the quality improvement program only funded sufficient medication review pharmacists to complete medication reviews in approximately 40% of high-risk patients.

### Participants

**Fig 1:**
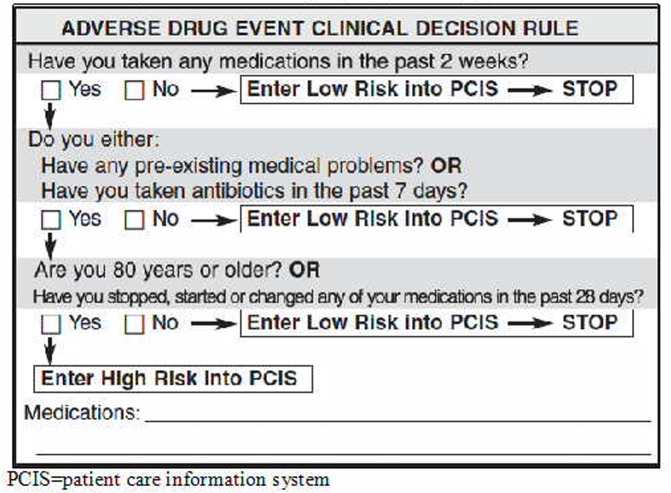
The modified Adverse Drug Event clinical decision rule used to identify patients at high-risk for adverse drug events in the emergency department. **[17]** PCIS = patient care information system.

Nurses identified incoming patients at high-risk of adverse drug events, based on the patients’ age, comorbid conditions, recent antibiotic use and recent medication changes at triage (Fig 1).[17] Consecutive high-risk patients aged 19 years or older presenting when a medication review pharmacist was on duty, were eligible for enrolment. We excluded patients requiring immediate resuscitation according to the Canadian Triage Acuity Score (CTAS), a five-level scale that allows triage nurses to prioritize patient care according to the acuity of a patient’s presentation (CTAS = 1).[18] We also excluded patients presenting for multisystem trauma, a scheduled re-visit (*e*.*g*., for intravenous antibiotics), sexual assault, postsurgical or pregnancy-related complication, social problems (*e*.*g*., those presenting without an acute medical problem), and those for whom we could not link to administrative records. We enrolled patients at their first eligible visit, and categorized them according to their treatment assignment on that visit.

### Ethical considerations

Prior to this study, emergency pharmacists were only able to assess a small fraction of patients during business hours on weekdays, creating a dual standard of pharmacy care at all participating sites. The supplementary funding provided during the quality improvement program precluded the provision of enough additional medication review pharmacists to provide the service to all eligible patients. As a result, creation of a control group of patients for the purposes of evaluation was deemed ethical. The University of British Columbia Clinical Research Ethics Board reviewed the protocol, deemed it quality improvement evaluation, and waived the need for full board review and informed consent.

### Intervention

The intervention, early in-hospital pharmacist-led medication review, consisted of obtaining a best-possible medication history, discussing the goals of therapy with the patient or caregiver, and reviewing the patient’s medications to identify and resolve medication-related problems, including adverse drug events, unintended and harmful events related to medication use, and optimize medication effectiveness and safety. We provided a mechanism in our electronic patient tracker to allow pharmacists to document the types of medication-related problems they encountered, according to Hepler & Strand’s definitions.[19] Because of the pragmatic nature of the intervention, and the real-world setting of our implementation, we did not proscribe any study definitions. The intervention was delivered by medication review pharmacists all of whom were residency-trained clinical pharmacists with a minimum of two years’ working experience in an acute care hospital. Medication review pharmacists were oriented to the program in a two-week training period during the second phase of piloting. These pharmacists had access to all hospital records, laboratory and diagnostic test results. They reviewed all suspected adverse drug events requiring treatment with the patients and their caregivers, the treating emergency or admitting physicians, and documented them in medical records. Medication review pharmacists attempted to contact the patients’ family or prescribing physicians and community pharmacists by telephone, if possible, and faxed notes to family physicians with their pager numbers on them. All medication review pharmacists carried pagers to facilitate communication with physicians and other staff.

We scheduled medication review pharmacists according to the influx patterns of high-risk patients during the pilot phase at each site, providing 8 hours per day of coverage on historically low-volume days, and up to 16 hours of coverage on historically high-volume days of the week. We allowed for double or triple coverage during high volume periods of the day (*e*.*g*., between 1pm and 9pm) while leaving low volume times (*e*.*g*., between midnight and 7am) uncovered.

### Control

Patients in the control arm received nurse or physician-led medication reconciliation using electronic forms that were pre-populated with the patient’s outpatient medication dispensing record. Medication reconciliation included obtaining a best possible medication history, and using this history as a basis for prescribing. Emergency pharmacists only assessed control group patients if a nurse or physician requested a consultation for a specific medication management problem. Ward-based pharmacists were able to complete medication reviews in the control group after patients were admitted to a ward on weekdays during business hours.

### Treatment allocation

Given the pay-for-performance reimbursement structure of the program and its aim to expand access to pharmacist-led medication review, we designed a group allocation algorithm that enabled medication review pharmacists to complete as many interventions as possible, while attempting to create two comparable groups.[16] Due to the variable influx of patients into, and the steady egress of lower acuity patients out of emergency departments, medication review pharmacists randomly selected one high-risk patient from among all high-risk patients who had presented within 60 minutes of the start of their data collection shift using an online random number generator. To maximize the number of interventions that they could complete during their shift (a requirement of the pay-for-performance reimbursement structure of the quality improvement program), enable follow-up of patients as their laboratory and diagnostic tests became available, and minimize incomplete interventions, the first randomly selected patient was always allocated to the medication review group. Subsequently, consecutive eligible patients were allocated to the medication review or control groups in pre-determined ratios of between 1:1 and 4:1. The allocation ratio was determined at the beginning of each shift based on the number of patients waiting to be triaged, in a manner that was blinded to patient characteristics. The flexible allocation ratio allowed us to adjust the number of interventions delivered to the volume of incoming patients while creating a control group, and enabled us to maximize the number of interventions and minimize incomplete interventions.

### Outcome measures

The primary outcome was the number of days spent in-hospital during the 30-day follow-up period, as contained in the Discharge Abstract Database (DAD). We picked this primary outcome and follow-up duration, as prior work showed an inconsistent effect of pharmacist-led medication reviews that focused on discharge interventions, on readmissions and repeat emergency department visits. Thus, we planned our intervention as early as possible after hospital arrival to evaluate its effect on the length of hospital stay. We knew from prior work that high-risk patients would have an average length of hospital stay of around 6 days, and wanted to capture the length of the first hospital stay, as well as hospital days incurred due to readmissions. Secondary outcomes included: emergency department re-visits within 7 days (National Ambulatory Care Reporting System [NACRS]), hospital length of stays exceeding the expected length of stay (eLOS) given the age and case mix group assignment (DAD), admissions (DAD), unplanned readmissions (DAD), and all-cause mortality (BC Vital Statistics).[20] Given the heterogeneity of our target population (undifferentiated patients presenting for unplanned admissions) and the inaccuracy of diagnostic medical codes, we used the eLOS variable validated by the Canadian Institutes of Health Information, that describes a patient’s expected length of stay given their age and case mix group category, to compare patients’ actual with their expected length of stay, allowing us to account for heterogeneity without restricting our analyses to few arbitrary patient strata. All study outcomes were determined in a manner that was blinded to treatment assignment by using patient-level anonymized linkage with administrative health databases.

### Statistical analysis

We summarized categorical variables using frequency distributions, and continuous variables using means with standard deviations or medians with interquartile ranges. We used median regression to estimate the association between medication review and the primary outcome, and logistic regression to estimate the association between medication review and secondary outcomes using control patients as the reference group. We adjusted the median regression model for baseline and healthcare access characteristics by including age, sex, socioeconomic status, number of medications, Canadian Triage Acuity Score, emergency department arrival time, emergency department arrival mode, weekday of presentation, and hospital crowding in the model. As the number of comorbid conditions was highly collinear with the number of medications taken, we controlled for comorbidity by controlling for the numbers of medications used as determined by linkage to outpatient medication dispensing data. We used these same variables to create inverse propensity scores to estimate the probability of assignment to the intervention or control groups, and used them to weigh each observation entered into the logistic regression analyses.[21, 22] We generated intervention effect estimates for each study site, and pooled them across sites using the *Der-Simonian Laird* random effects model.[23] We conducted *a priori* defined subgroup analyses using age 80 as a cutoff for the number of days in hospital. We used ages 60 and 80 as cutoffs according to the case mix grouping methodology for analyses on the expected length of stay.[20] Estimates were presented with 95% CIs. The sample size was a function of the duration of the quality improvement program.

## Results

### Treatment assignment and baseline characteristics

**Fig 2:**
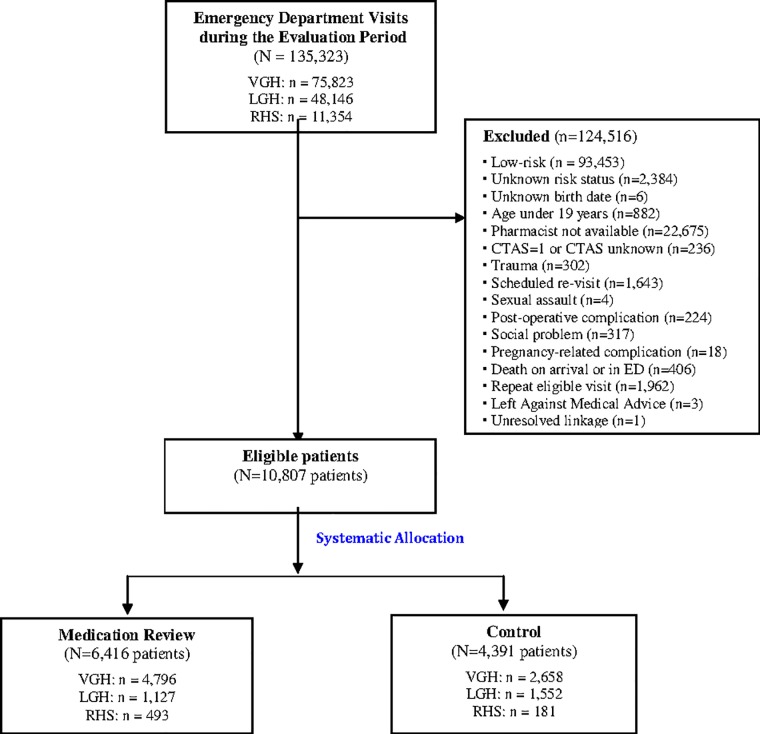
Flow diagram of patients through the study. VGH: Vancouver General Hospital; LGH: Lions Gate Hospital; RHS: Richmond Hospital; ED: emergency department; CTAS: Canadian Triage Acuity Score.

During the Adverse Drug Event Screening Program, 135,323 patients presented to the emergency departments of all participating sites. Patients were most commonly excluded because they were classified as low-risk for adverse drug events (n = 93,453), or because no medication review pharmacist was on shift when they presented (n = 22,675). We enrolled 10,807 high-risk patients, of whom 6,416 received medication review in the emergency department, and 4,391 usual care (Fig 2). The groups were balanced with regard to age, sex, number of medications, triage acuity, and socioeconomic status. The medication review group had a higher proportion of patients who presented by ambulance, indicating higher acuity at presentation (Table 1).

**Table 1:** Baseline characteristics of enrolled patients, by group assignment.

	Medication Review(n = 6,416)	Control (n = 4,391)	p-values
**Age—yrs**	71±31	69±33	0.006
**Male sex—no. (%)**	2,797 (43.6%)	1,969 (44.9%)	0.196
**Medications**	8.1±5.9	7.7± 5.9	0.001
**Most common chief complaint—no. (%)**			0.335
Chest Pain	698 (10.9%)	650 (14.8%)	
Abdominal Pain	428 (6.7%)	319 (7.3%)	
Shortness of Breath	312 (4.9%)	187 (4.3%)	
**Arrival mode—no. (%)**			<0.001
Ambulance	2,381 (37.1%)	1,442 (32.9%)	
Walk-in	3,735 (58.2%)	2,550 (58.1%)	
Other	300 (4.7%)	397 (9.0%)	
**Canadian triage acuity score—no. (%)**			0.454
Emergent (category 2)	1,487 (23.2%)	1,069 (24.4%)	
Urgent (category 3)	3,742 (58.3%)	2,510 (57.2%)	
Semi Urgent (category 4)	1,135 (17.7%)	769 (17.5%)	
Non Urgent (category 5)	52 (0.8%)	41 (0.9%)	
**Median household income—no. (%)**			<0.001
First (lowest) quintile	753 (11.7%)	493 (11.2%)	
Second quintile	1,466 (22.8%)	832 (18.9%)	
Third quintile	1,100 (17.1%)	721 (16.4%)	
Fourth quintile	1,139 (17.8%)	915 (20.8%)	
Fifth quintile	1,878 (29.3%)	1,369 (31.2%)	

### Intervention details

In the medication review group, pharmacists identified adverse drug events in 2,284 (35.6%) high-risk patients. Pharmacists classified the events as adverse drug reactions in 630 patients (27.6%), untreated indications in 546 (23.9%), non-adherence in 423 (18.5%), ineffective or wrong drug in 284 (12.4%), subtherapeutic dose in 201 (8.8%), supratherapeutic dose in 124 (5.4%), and unnecessary drug in 76 (3.3%).

### Primary outcome

Outcome data on all patients were complete. The median number of hospital days was reduced by 0.48 days (95% confidence interval [CI] = 0.00 to -0.96; p = 0.058) in the medication review group compared to the control group (Table 2), representing an 8% relative reduction in the number of days spent in hospital over the follow-up period. This difference was more pronounced among patients under the age of 80, who spent a median of 0.60 fewer days (95% CI = 0.06 to 1.17; p = 0.03) in-hospital, representing an 11% relative reduction in the number of days spent in hospital during follow-up. In patients over the age of 80, medication review had no effect on the median number of hospital days (-0.06 days; 95% CI = -0.90 to 0.78; p = 0.88).

**Table 2:** Primary and Secondary Outcomes, and Treatment Effects.

Outcome	Intervention (n = 6,416)	Control (n = 4,391)	Effect Variable	Unadjusted Value (95% CI)	Adjusted Value§ (95% CI)
**Primary Outcome:** Days in-hospital within 30 days, among admitted median (interquartile range)*	5.67 (2.69 to 12.70)	5.79 (2.64 to 12.79)	Median difference	-0.12 (-0.30 to 0.06)	-0.48 (-0.96 to 0.00)
**Secondary Outcomes:**					
Emergency department revisits within 7 days—no. (%)†	414	310	Odds ratio	0.91 (0.78 to 1.06)	1.01 (0.84 to 1.22)
Hospital admission—no. (%)	2,549	1,698	Odds ratio	1.05 (0.97 to 1.13)	0.98 (0.90 to 1.06)
Length of stay exceeding expected length of stay, by age category—no. (%)*	1089	726	Odds ratio	0.99 (0.88 to 1.13)	0.91 (0.80 to 1.03)
18 to 59 years	257	194	Odds ratio	1.06 (0.83 to 1.36)	1.03 (0.79 to 1.34)
60 to 79 years	310	221	Odds ratio	0.86 (0.69 to 1.08)	0.73 (0.57 to 0.92)
>80 years	522	311	Odds ratio	1.06 (0.88 to 1.28)	1.00 (0.82 to 1.22)
Unplanned readmissions, among admitted—no. (%)‡	206	154	Odds ratio	0.90 (0.71 to 1.13)	0.87 (0.69 to 1.10)
Mortality—no. (%)	492	311	Odds ratio	1.09 (0.94 to 1.26)	1.11 (0.96 to 1.30)

* The outcome was calculated based on patients who were admitted to hospital on the index emergency department visit date (n = 2500 in the intervention, and n = 1,668 in the control group). Sixteen patients with missing data on socioeconomic status were excluded from the propensity score modeling.

† Emergency department revisits were calculated based on patients who were discharged from the emergency department on the date of the index visit (n = 3,914 in the intervention, and n = 2,696 in the control group).

‡ Unplanned readmissions were calculated based on patients who were admitted on the index emergency department visit and discharged from hospital within the follow-up period (n = 2,430 in the intervention, and n = 1,619 in the control group).

§ Propensity score models predicted treatment assignment based on the variables age, sex, socioeconomic status, number of medications, Canadian Triage Acuity Score, Emergency Department arrival time, Emergency Department arrival mode, weekday of presentation, and hospital crowding.

### Secondary outcomes

Among patients discharged from the emergency department, 414 of 3,914 (10.6%) who received medication review revisited the emergency department within 7 days, compared to 310 of 2,696 (11.5%) in the control group. After controlling for confounding variables, medication review had no effect on the odds of emergency department revisits (odds ratio [OR] = 1.01, 95% CI = 0.84 to 1.22; p = 0.88; Table 2). In the medication review group, 2,549 of 6,416 (39.7%) of patients were admitted to hospital compared to 1,698 of 4,391 (38.7%) in the control group. After controlling for baseline characteristics, medication review had no effect on the odds of being admitted to hospital (OR = 0.98; 95% CI **=** 0.90 to 1.06; p = 0.55; Table 2).

To account for the heterogeneity in admitting diagnoses and comorbidity levels among high-risk patients, we compared actual with expected lengths of stay for admitted patients, given their case mix group. Among patients between 60 and 79 years of age, fewer patients in the medication review group (310 of 777; 39.9%) exceeded the expected length of stay compared to control (221 of 508; 43.5%). The odds of exceeding the expected length of stay in those aged 60–79 were 0.73 (95% CI = 0.57 to 0.92; p = 0.01) in the medication review group compared to control (Table 2). We found no differences for other age groups. Among patients admitted and subsequently discharged from hospital, 206 of 2,430 patients (8.5%) were readmitted in the medication review group compared to 154 of 1,619 (9.5%) in the control group. After controlling for baseline characteristics, medication review had no effect on the odds of being readmitted (OR = 0.87; 95% CI = 0.69 to 1.10; p = 0.25; Table 2). In the medication review group 492 of 6,416 patients died (7.7%) within the follow-up period compared to 311 of 4,389 in the control group (7.1%). After adjustment, there was no difference in mortality (OR = 1.11; 95% CI = 0.96 to 1.30; p = 0.16; Table 2).

## Discussion

The implementation of the Adverse Drug Event Screening Program in three hospitals provided an opportunity to evaluate the effect of early in-hospital medication review on high-risk patients. We found a trend, but no statistically significant effect on hospital days in high-risk patients with access to early pharmacist-led medication review. Among patients less than 80 years of age, the reduction was more pronounced and statistically significant. The relative length of stay reduction among high-risk patients in this age group was felt to be clinically significant, as it amounted to a reduction in the length of day by over half a day per high-risk patient who received the intervention. As overcrowded hospitals increasingly discharge patients on weekends and into the evenings this degree of length of stay reduction is significant, and resulted in cost savings that fully funded the pharmacists’ salary in participating hospitals.

Until now, few studies have evaluated the effect of in-hospital medication review on downstream health services use in undifferentiated patients such as those with unplanned admissions to hospital.[15, 24] Most were conducted in Europe,[14, 25–31] enrolled limited numbers of patients [14, 25, 26, 28–34] and did not provide medication reconciliation to patients in the control arm, the current standard in North America. All but one study evaluated interventions delivered by a maximum of three pharmacists with varied levels of training and experience, making it difficult to generalize the interventions outside of the study settings.[27] Interventions were generally delivered only on weekdays and during business hours, and after patients had been admitted to hospital wards, thus delaying the time to appropriate medication therapy and minimizing its impact on length of stay, especially for off hours and weekend admissions. Two systematic reviews summarizing the results of these studies have found no impact on the length of hospital stay, readmissions, and mortality.[15, 24] Yet, most acute care hospitals in North America provide medication review services to admitted patients.

We evaluated the effect of medication review in a cohort of carefully selected high-risk patients who received the intervention in the emergency department, thus earlier within a patient’s hospital course than previous studies, and making medication review results available during the admission process. We employed trained medication review pharmacists with at least 2 years of experience in acute care settings to deliver the medication review intervention during high volume times of the day/evening and week. Patients in the control arm received medication reconciliation by a nurse or physician and had access to emergency pharmacists for specific medication management questions, and therefore, received the current standard of pharmacy care in North America. We selected outcome measures that were recorded by assessors who were blinded to group assignments, because study participants and healthcare providers could not be blinded to the medication review intervention, as the effect of medication review is mediated by changes in decision-making. While ward-based clinicians had access to the medication review results while making discharge decisions, they were unaware of the ongoing evaluation and its outcome measures. We incorporated any days spent in-hospital within the follow-up period into the primary outcome measure to ensure that in the case of inappropriate early discharges subsequent readmit days were captured.

Our evaluation results reflect one tertiary care, and two urban community hospitals, one of which has a focus on geriatric care. Therefore, our results reflect a heterogeneous sample of high-risk patients, and are likely generalizable to other urban acute care hospitals of similar size. As we were unable to adjust for alternate level of care days among older frail adults, it is possible that the effect of the intervention among older adults was diluted, as overall frailty and inability to ambulate independently or care for themselves may have been the primary determinants of their length of stay, thus diluting the signal to noise ratio.

### Limitations

Our evaluation is not without limitations. Defining the most appropriate outcome measure for medication review was challenging. We expected the intervention to add diagnostic information about adverse drug events resulting in drug therapy changes, and a maximal treatment effect in patients with otherwise undiagnosed events.[9, 10, 35] Ideally, we would have captured the treatment effect in this patient group. However, it is impossible to identify undiagnosed adverse drug events in control patients without reviewing their medications, and it would be unethical not to treat them once diagnosed. Thus, we compared high-risk patient groups in which the events were concentrated to improve the signal to noise ratio. Lack of reliable identification of adverse drug events within administrative data, and within medical charts precluded accurate measures of adverse drug event-related emergency department revisits or hospitalizations.[36]

We systematically allocated patients to treatment groups to minimize selection bias and incomplete interventions, as the pay-for-performance structure of the program precluded randomization. While this falls short of the methods used in randomized trials to ensure balance between treatment groups, the groups appeared to be well balanced, and the use of propensity score modeling is likely to have reduced the effect of any imbalances. Despite this, residual and unknown confounding could have biased the treatment effect. Finally, blinding to group allocation is not possible in medication review evaluation as the review results must impact clinical decision-making. However, we believe it unlikely that lack of blinding led to an overestimation of the treatment effect because the healthcare providers on wards making discharge decisions were unaware of the ongoing evaluation and its outcomes, and the medical coders ascertaining and recording outcomes in administrative data were blinded to treatment allocation. It is possible that contamination between the control and intervention groups occurred as care providers could have incorporated aspects of medication review into their practice, making it more difficult to find differences between groups. As this was a pragmatic evaluation of a real-world quality improvement program, we were unable to document the fidelity of pharmacists delivering the intervention, or Finally, we were unable to adjust for ward-based medication review interventions that were completed after patients left the emergency department, as these were not documented within the administrative data.

### Lessons

Early pharmacist-led medication review in high-risk emergency department patients was associated with a trend towards reduced hospital-bed utilization, but was only statistically significant in patients under the age of 80. The results of our evaluation may be used to guide pharmacist-led medication review interventions in acute care hospitals, and suggests that targeting specific patient populations may be important. Given the limitations of our methodology, a randomized control trial on early medication review should confirm the effect of the intervention, with particular attention to its effectiveness in various age groups.
